# Approach to the Patient: Low Testosterone Concentrations in Men With Obesity

**DOI:** 10.1210/clinem/dgaf137

**Published:** 2025-03-07

**Authors:** Christopher A Muir, Gary A Wittert, David J Handelsman

**Affiliations:** Department of Andrology, Concord Repatriation General Hospital, Sydney, NSW 2139, Australia; Faculty of Medicine and Health, University of Sydney, Sydney, NSW 2050, Australia; School of Clinical Medicine, University of New South Wales, Sydney, NSW 2052, Australia; South Australian Health and Medical Research Institute, Adelaide, SA 5000, Australia; Freemasons Centre for Male Health and Wellbeing, University of Adelaide, Adelaide, SA 5000, Australia; Department of Andrology, Concord Repatriation General Hospital, Sydney, NSW 2139, Australia; ANZAC Research Institute, University of Sydney, Sydney, NSW 2138, Australia

**Keywords:** obesity, testosterone, hypogonadism, androgen deficiency

## Abstract

Pathologic hypogonadism occurs when serum testosterone is significantly and persistently reduced by irreversible organic (structural, genetic) disorders of the hypothalamic pituitary testicular axis. Men with pathologic hypogonadism require lifelong testosterone replacement. In contrast, mild or moderate reductions in serum testosterone frequently accompany obesity, and its numerous comorbidities in men are best considered nongonadal illness syndromes, wherein reduction in serum testosterone is usually reversible upon amelioration of the underlying nongonadal illness.

Obesity can result in nonspecific symptoms in conjunction with reduced serum testosterone and serum SHBG. Obesity-related reductions in SHBG, testosterone's principal circulating carrier protein, are primarily responsible for measured reductions in testosterone. However, obesity is not a cause of pathological hypogonadism, and proportionately reduced testosterone and SHBG concentrations accompanied by normal serum LH and FSH concentrations confirm a eugonadal state, best described as the pseudo-hypogonadism of obesity.

Herein we demonstrate how clinically significant weight loss substantially reverses obesity-related reductions in serum testosterone and ameliorates nonspecific symptoms resembling, but not due to, androgen deficiency. The important reversible steps include weight reduction and optimizing management of type 2 diabetes mellitus, obstructive sleep apnea, depression, and other obesity-related comorbidities as well as rationalizing concomitant drug regimens. In the absence of pathological hypogonadism, testosterone treatment is less effective than a diet and lifestyle intervention to rectify the reversible conditions responsible for the nonspecific symptoms and associated reduced serum testosterone concentrations observed in men with obesity. As such, testosterone treatment is not indicated, and unwarranted off-label testosterone treatment can lead to adverse effects such as infertility, elevated hematocrit requiring venesection, a prothrombotic state, and testosterone dependence.

Key Points

- Serum testosterone concentration is often reduced in men with obesity, proportional to the degree of excess weight.
- In men with obesity, low serum testosterone concentrations alone do not equate to a diagnosis of hypogonadism, even if nonspecific symptoms resembling testosterone deficiency are present.
- Reduced serum testosterone concentrations in men with obesity are predominantly due to obesity-related effects lowering serum SHBG. Serum LH and FSH operate as highly sensitive tissue androgen sensors and remain normal, indicating the pseudo-hypogonadism of obesity is a eugonadal state.
- In men with obesity and low serum testosterone concentrations but without pathological hypogonadism, treatment should focus on identification and rectification of obesity related comorbidities, optimizing health-related lifestyle behaviors and interventions to promote significant and sustained weight loss without risking the adverse effects of unjustified testosterone treatment.

Mild to moderate reduction in serum testosterone concentrations with typically normal serum LH and FSH are frequent in men with overweight/obesity. Such men may also complain of nonspecific symptoms resembling those seen in androgen deficiency or from many other age-related comorbidities (1). This phenomenon has been termed the pseudo-hypogonadism of obesity, which reflects that the combination of nonspecific symptoms and lowered serum testosterone concentrations does not equate to an organic state of hypogonadism and as such testosterone replacement therapy is not justified (2). Obesity-related pseudo-hypogonadism is a common variant of the nongonadal illness syndrome, a variety of nongonadal conditions that cause a modest lowering of serum testosterone as a nonspecific and potentially reversible barometer of general health but retaining a eugonadal state for which testosterone treatment is not justified.

There is no evidence that men with simple obesity have pathologic hypogonadism understood as structural or genetic disorders of the hypothalamus, pituitary, or testes. Clinical trial data in older men provide little evidence to suggest substantial health benefit(s) from testosterone treatment in contrast to even modest lifestyle interventions (3-5). Using an example of a patient from our clinic, we outline a framework for assessing and managing the increasingly prevalent finding of pseudo-hypogonadism involving a mild to moderate lowering of serum testosterone concentrations with preservation of normal serum LH and FSH in men with overweight/obesity.

## Case Presentation

A 37-year-old analyst was referred for evaluation of low serum testosterone levels. He described several years of progressive fatigue, reduced libido, erectile dysfunction, poor recovery after exercising, and difficulty losing weight. Additionally, he had been diagnosed with obstructive sleep apnea (OSA) and hypertension, treated with continuous positive airway pressure and candesartan 8 mg daily, respectively. On the advice of a coworker and after conducting online “research,” he requested serum testosterone testing by his primary care physician, which returned a result of 147 ng/dL (5.1 nmol/L), below the laboratory lower reference interval. He was then referred for review of “testosterone deficiency” and consideration of testosterone replacement therapy.

He described longstanding obesity with a current weight of 118 kg [body mass index (BMI) 36 kg/m^2^] and predominant central adiposity (waist circumference 112 cm; normal range, NR <94 cm). Resting blood pressure was elevated (148/99 mmHg). Virilization and secondary sexual characteristics were normal with testicular volumes of 25 mL bilaterally (Prader orchidometer). Repeat early morning blood sampling confirmed low serum testosterone with concomitant low SHBG and normal LH and FSH concentrations (Table 1). Additional obesity-related metabolic abnormalities were also present (Table 1). Hemoglobin and hematocrit were within the normal range.

**Table 1.** Laboratory data for case patient

Variable	Baseline result	Follow-up result	Reference range
Weight (kg)	118	94	
BMI (kg/m^2^)	36.0	28.7	
Testosterone*^a^* (ng/dL)	147	467	231-808
	(5.1 nmol/L)	(16.2 nmol/L)	(8-28 nmol/L)
SHBG*^b^* (nmol/L)	14	33	12-50
LH*^c^* (IU/L)	2.1	3.8	1-8
FSH*^d^* (IU/L)	3.2	3.6	1-8
Fasting glucose (mg/dL)	120	93	70-100
HbA1c (%)	6.2	5.4	<5.9
ALT (U/L)	107	18	0-40
AST (U/L)	56	20	0-35
Total cholesterol (mmol/L)	5.0	4.3	<5.5
LDL cholesterol (mmol/L)	3.3	2.8	<3.0
HDL cholesterol (mmol/L)	1.2	1.2	>1.0
Triglycerides (mmol/L)	2.3	1.7	<2.0

Abbreviations: ALT, alanine aminotransferase; AST, aspartate aminotransferase; BMI, body mass index; HbA1c, hemoglobin A1c; HDL, high-density lipoprotein; LDL, low-density lipoprotein.

^*a*^Roche Cat# 07027915190, RRID: AB_3101983.

^*b*^Roche Cat# 03052001190, RRID: AB_2891222.

^*c*^Roche Cat# 07027575190, RRID: AB_2920601.

^*d*^Roche Cat# 07027346190, RRID: AB_2920600.

## Background

Overweight and obesity are risk factors for a large number of chronic diseases spanning numerous organ systems, and improvement in organ function following clinically significant weight loss is well documented (6). The effects of obesity on testicular function are also well known, with reductions in circulating testosterone concentration strongly associated with increasing visceral adiposity and metabolic syndrome (7). Although this relationship is likely bidirectional, by far the strongest relationship is via the effect of obesity on circulating testosterone levels, with much smaller effects of low testosterone on increasing adiposity (8). Incorrectly, lay media and many doctors consider a “low” testosterone concentration in isolation as observed in men with overweight and obesity as constituting a form of hypogonadism. That implies the “low” testosterone is mechanistically responsible for the obesity and that treatment with testosterone is indicated, despite no well-conducted studies suggesting the efficacy or safety of testosterone treatment in this clinical setting (2). Furthermore, pseudo-hypogonadism of obesity is a major contributor to the quasi-epidemic of testosterone misuse, with a 100-fold increase in testosterone prescribing over 3 recent decades without any new approved indications (9).

### Obesity Effects on the Hypothalamic-pituitary-testicular Axis

There is no high-quality evidence supporting obesity as a cause of pathological hypogonadism. Although a strong inverse association exists between the degree of obesity and circulating testosterone concentrations, a much weaker relationship is observed when comparing the relationship between obesity and testosterone in the opposite direction (10). This stark contrast is best highlighted in men with prostate cancer undergoing androgen deprivation therapy, whereby serum testosterone concentrations are reduced to castrate levels with minimal increase (2.4%) in underlying body weight (11).

In most men with simple obesity, the mild to moderate reduction in serum testosterone is mediated through the lowering of circulating SHBG due to adiposity-associated metabolic abnormalities such as hyperinsulinemia, hypertriglyceridemia, and hepatic steatosis (10, 12).

Despite the reduction in serum testosterone concentration, stimulation of testis and pituitary gland function using human chorionic gonadotropin and gonadotropin-releasing hormone, respectively, prove that both organs are dynamically responsive, consistent with a eugonadal state (8). Reduction in LH pulse amplitude (but not pulse frequency) has been observed in men with class III obesity (BMI >40 kg/m^2^), suggesting that extreme obesity may have a modest impact on the hypothalamic-pituitary-testicular (HPT) axis function (13). The consistent presence of normal serum LH and FSH, acting as tissue androgen sensors, like how TSH operates for thyroid status, further supports that simple obesity is a eugonadal state. However, like the decrease in serum testosterone concentration itself, such changes are responsive to weight loss and do not constitute pathological hypogonadism due to irreversible structural or genetic causes of impaired HPT axis function.

Studies examining alterations in estradiol, proinflammatory cytokines, or gastrointestinal hormones on testosterone concentrations in obese men show modest associations but have not demonstrated a convincing causal relationship sufficient to explain the consistent and proportionate lowering of serum testosterone and SHBG concentrations (8). For instance, a randomized trial of the anti-inflammatory IL-1 antagonist anakinra increased serum testosterone by a statistically significant but minimally impressive 34 ng/dL (1.2 nmol/L) compared to placebo (14). Similarly, markers of insulin resistance are often present in men with obesity and can improve with testosterone treatment due to treatment-related improvements in body composition (4). However, the reverse is generally not true, and treatment with an insulin-sensitizing medication such as metformin does not significantly increase testosterone concentrations (15, 16).

### Free Testosterone

Free testosterone, whether measured or calculated using equilibrium binding or empirical equations, is advocated by some experts to appraise androgen status in certain circumstances (17-19). However, such recommendations are based on observational data and received wisdom rather than objective evidence from controlled clinical testing and therefore in our opinion add nothing useful to the clinical evaluation (2, 20). The free testosterone hypothesis, which asserts that free testosterone is the most biologically active moiety of testosterone, is conceptually flawed in that unbound testosterone is equally accessible to sites of degradation as it is to target tissues, so there is no logical basis to claim it is more (rather than less) biologically active (20). In addition, reference laboratory dialysis-based measurements of free testosterone are nonrobust, costly, and rarely available. Most laboratories report calculations based on formulae incorporating serum testosterone and SHBG measurements. However, such calculations are consistently inaccurate relative to dialysis-based measurements due to assumptions such as using SHBG mass as a substitute for SHBG binding capacity, the use of 4 different nonharmonized SHBG immunoassays, and the use of 5 different empirical estimates of testosterone-SHBG binding affinity (20). Furthermore, free testosterone is not a valid analytical variable, as it lacks a certified reference standard, violating a fundamental principle of analytical chemistry requiring comparison of like with like. As a result, free testosterone lacks sufficient quality controls required to form reference ranges or compare results from 1 center/laboratory to another.

### Significance of Obesity-related Comorbidities

OSA is highly prevalent in men with obesity and is consistently associated with lower concentrations of serum testosterone and SHBG compared to men without OSA. Decreases in testosterone appear to be mostly restricted to men with severe OSA, whereby mild and moderate OSA is not associated with significant perturbations in serum testosterone (21). It remains controversial whether successful treatment of OSA with continuous positive airway pressure restores serum testosterone to normal, with some studies demonstrating benefit but most showing no effect (22-25).

On average, serum testosterone concentrations are reduced by 57 to 86 ng/dL (2 to 3 nmol/L) in men with type 2 diabetes mellitus (T2DM) compared to nondiabetic controls (26, 27). The effect of T2DM on circulating testosterone is also independent of age and BMI and is consistent across different patient populations (27). Weight loss in overweight or obese men with T2DM effectively increases serum testosterone in proportion to the loss of excess weight; however, there is no evidence that improvements in glycemic control without concomitant weight loss have significant beneficial effects on testosterone concentrations (27, 28).

Testosterone levels are reduced in metabolic dysfunction-associated hepatic steatosis, advanced renal impairment, cardiovascular disease, and many other age- and weight-related comorbidities overrepresented in association with obesity (29). These conditions also entrain nonspecific symptoms indistinguishable from those of genuine testosterone deficiency. In obese men with OSA or T2DM and reduced serum testosterone, testosterone treatment is not indicated, as neither OSA nor T2DM are states of pathologic hypogonadism. Instead, the primary therapeutic intervention should include lifestyle modification, health optimization, and loss of excess weight, which can effectively reverse the nonspecific symptoms and reduced serum testosterone concentrations, proportionally to the degree of reduction in excess weight (8).

### Clinical Assessment and Pitfalls of Test Interpretation

Careful clinical assessment is required to avoid missing genuine cases of pathologic hypogonadism and to identify other conditions that reduce circulating testosterone concentration that may rarely occur by coincidence in men with overweight and obesity (ie, Klinefelter's syndrome, prolactinoma, hemochromatosis). History of cryptorchidism, testicular trauma, torsion or infection, and gonadotoxic exposures (ie, chemotherapy or radiotherapy) should be sought. Waist circumference and BMI are easily measured and useful to estimate the degree of central adiposity and obesity, respectively and proportionally. Testicular examination is mandatory, as normal testicular volume and consistency is generally inconsistent with the presence of pathologic hypogonadism. There is no basis to routinely screen for a low serum testosterone unless there is evidence to suggest undiagnosed pathologic hypogonadism including undervirilization (gynecomastia, eunuchoid proportions, loss of body hair) or reduced or atrophic testes. Measurement of testosterone can be considered in situations of marked acute diminution in sexual desire or unexplained anemia but is unlikely to be causally related unless severely reduced. However, in many instances serum testosterone will have been measured in primary care prior to referral. In such settings, it is important to appraise the full complement of serum testosterone, LH, FSH, and SHBG, as a fasting early morning sample (after at least 6 hours of restful sleep, notably for shift workers), ideally more than once as up to 30% of cases will spontaneously normalize on repeat testing (30). When testosterone concentrations are reduced, prolactin, TSH and iron studies should be tested to exclude reversible nongonadal illness syndromes that can rarely co-occur in men with obesity.

In most men with obesity, measured serum testosterone will be only modestly reduced, but very low concentrations can occur particularly in men with extreme obesity (eg, BMI >40 kg/m^2^). In such cases, the presence of a low serum testosterone, with proportionately low serum SHBG and normal-range serum LH and FSH (typical features of the pseudo-hypogonadism of obesity) in conjunction with a normal testicular examination is reassuring. These findings suggest that the hormonal aberrations are likely reversible due to obesity and will improve with significant weight loss. Magnetic resonance imaging of the pituitary and measurement of other anterior pituitary hormones is only required if specific additional clinical signs are present (ie, headache, galactorrhea, bitemporal hemianopia), if serum LH and FSH are very low or undetectable, or when serum testosterone concentration does not increase following clinically significant weight loss (31). Key features distinguishing cases of pathological hypogonadism from the pseudo-hypogonadism of obesity are summarized in Table 2.

**Table 2.** Distinguishing factors between pathological hypogonadism and the pseudo-hypogonadism of obesity

	Pathological hypogonadism	Pseudo-hypogonadism of obesity
Pathology	Organic (structural, genetic)	Functional (nongonadal illness syndrome)
Reversible	No	Yes
Testosterone	Reduced (moderate-severe)	Reduced (mild-moderate)
LH/FSH	Elevated (primary hypogonadism) or low/undetectable (secondary hypogonadism)	Normal (eugonadal)
SHBG	Normal	Reduced
Testis volume	Reduced; atrophic consistency (if acquired postpubertally)	Normal
Virilization	Reduced	Normal
Testosterone treatment	Required, lifelong	No

### Treatment Recommendations

**Figure 1.**
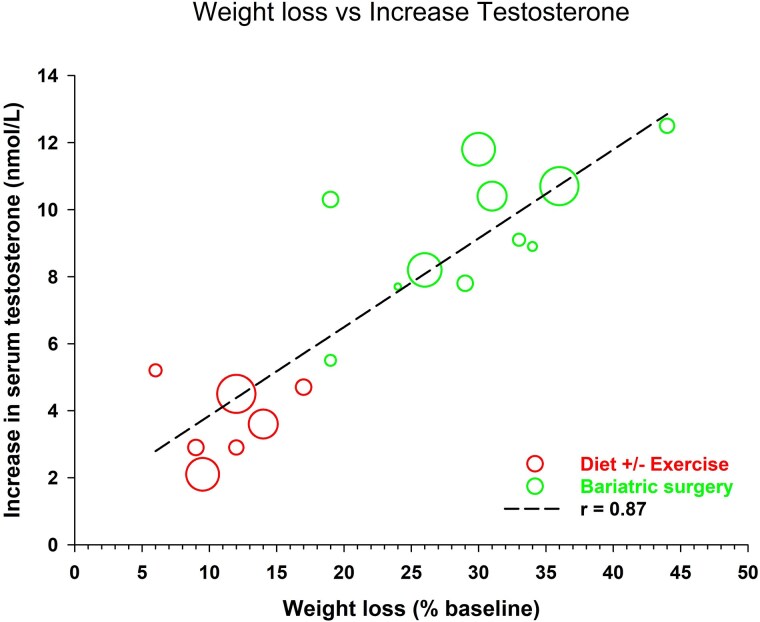
Relationship between weight loss achieved through diet, exercise, or bariatric surgery and resultant absolute increase in testosterone concentration. Adapted from Grossmann M. Low testosterone in men with type 2 diabetes: significance and treatment. J Clin Endocrinol Metab. 2011;96(8):2341-2353 with additional data from Grossman M, Matsumoto AM. A perspective on middle-aged and older men with functional hypogonadism: focus on holistic management. J Clin Endocrinol Metab. 2017;102:1067-1075.

Testosterone is not indicated for treatment of reduced serum testosterone concentrations in men with overweight and obesity, except where there is also pathological hypogonadism, which should be excluded by history, physical examination, and relevant laboratory testing. In simple obesity, weight loss is critical to restoring health, with meta-analyses on the effect of diet, exercise, and bariatric surgery showing marked increases in serum testosterone concentrations proportionate to the degree of weight loss achieved (Fig. 1) (26, 27, 32).

Serum testosterone and SHBG increase significantly but gradually following the implementation of a very low calorie diet (33). Improvements in serum testosterone and SHBG concentrations are maintained long term after a very low calorie diet-induced weight loss, so long as there is not substantial weight regain (34). Importantly, serum testosterone concentrations should not be retested during the dynamic phase of energy restriction, as testosterone concentrations may temporally decrease and will only improve sustainably once the nutrient intake is more balanced and weight loss has plateaued. Physical activity, particularly in conjunction with a calorie-reduced dietary intervention, significantly increases serum testosterone concentrations and improves body composition (35). In the Diabetes Prevention Program trial including more than 1000 overweight or obese men at risk for T2DM randomized to placebo, metformin, or a lifestyle intervention, only the intensive lifestyle intervention (including 150 minutes of physical activity per week) was associated with an increase in serum testosterone concentration after an average 2.8 years of follow-up (16). Bariatric surgery such as gastric banding, sleeve gastrectomy, and gastric bypass also produce rapid and sustained weight loss with consequent increases in serum testosterone proportional to the amount of weight lost (36), although effects on sperm production appear less beneficial (37, 38).

Much less is known on the effects of glucagon-like peptide-1 (GLP-1) receptor agonists, and there are no data on newer medications containing GLP-1 receptor agonists in combination with other incretin hormones on the pseudo-hypogonadism of obesity (39). As the use of such medications expands exponentially for the management of chronic obesity, their full impact on testicular endocrine (testosterone) and exocrine (spermatogenesis) function needs to be clarified. The ability of GLP-1-containing antiobesity medications to restore serum testosterone concentrations to those comparable with normal weight men may be a potential benefit of these therapies that remains to be proven.

Taken together, data suggest that weight loss irrespective of how it is achieved is highly effective in reversing the functional reduction in serum testosterone observed in men exhibiting pseudo-hypogonadism of obesity, particularly when other obesity-related comorbidities such as OSA and T2DM are present.

An important but little appreciated consequence of testosterone treatment for men without pathological hypogonadism is the risk of androgen dependence. In men with pseudo-hypogonadism, exogenous testosterone use suppresses the HPT axis, creating a sustained reduction of endogenous testosterone production, which can be slow to reverse after cessation. By contrast, men with pathologic hypogonadism have minimal residual endogenous testosterone production and require lifelong testosterone replacement therapy. Androgen dependence is a well-recognized consequence of androgen misuse and abuse whereby exogenous testosterone intake leaves men with acquired, symptomatic androgen deficiency when intake ceases, even when they are not androgen deficient before the start of androgen abuse. The recovery period can be prolonged (6-18 months), during which time androgen deficiency withdrawal symptoms can drive men to resume androgen intake, resetting the recovery clock to zero and creating a vicious cycle of androgen dependence. These symptomatic effects of withdrawal from exogenous androgens are related to the time since stopping androgen intake and not the dose, duration, or type of androgens abused (40, 41). Similar, though less frequent, effects have also been reported with testosterone administration at physiologic replacement doses in men without pathological hypogonadism (42). The consequences of androgen dependence are comparable to those of glucocorticoid dependence when pharmacological doses are used in the absence of adrenal failure.

## Back to the Patient

The patient was counseled on healthy eating and adherence to a calorie-controlled diet. He was referred to a dietician and exercise physiologist for additional assistance in developing a personalized diet and exercise plan. Over the following 6 months, weight reduced slowly to a nadir of 107 kg (BMI 32.7 kg/m^2^). Despite continued adherence to a diet and lifestyle intervention, weight loss plateaued. At this stage, he was commenced on semaglutide, uptitrated to a dose of 2.4 mg weekly. Following the introduction of semaglutide, he experienced further weight loss, achieving a nadir weight of 94 kg (BMI 28.7 kg/m^2^) after 12 months of treatment. With weight loss, fatigue and other symptoms reported at baseline improved. Resting blood pressure normalized, and candesartan was successfully withdrawn. Laboratory testing was repeated and demonstrated a marked increase in serum testosterone and SHBG with no change in gonadotropin concentrations. Similar improvements were observed in other metabolic markers of health (Table 1).

## Conclusion

Our case illustrates the confounding effect of obesity on the measurement of circulating testosterone in men. It is important for clinicians to understand the limitations of interpreting an isolated low serum testosterone and the need to avoid assuming that a low serum testosterone concentration equates to a diagnosis of androgen deficiency without evidence of concomitant pathologic hypogonadism. Therefore, in the absence of such evidence, there is little use in screening for androgen deficiency in men with obesity, even if nonspecific symptoms are present. Clinically significant weight loss using diet, lifestyle, medical, and/or surgical interventions can produce substantial increases in measured serum testosterone and are the only appropriate treatment modalities. Treatment with testosterone should be avoided in men with obesity without pathologic hypogonadism, as the proposed benefits and safety are unproven in addition to the known adverse effects of testosterone treatment on spermatogenesis and the potential for androgen dependence.

## Data Availability

Data sharing is not applicable to this article as no datasets were generated or analysed.
